# Supplementary material for: Blood-Based Liquid Biopsy for Comprehensive Cancer Genomic Profiling Using Next-Generation Sequencing: An Emerging Paradigm for Non-invasive Cancer Detection and Management in Dogs
Source: Front Vet Sci. 2021 Jul 8;8:704835. doi: 10.3389/fvets.2021.704835 (PMC8297996; doi:10.3389/fvets.2021.704835)
Supplement: Supplementary file 1 [file Table_1.pdf]

SUPPLEMENTARY TABLE 1. *Full subject-level genomic results.* Genomic profiling identified somatic alterations in tissue from all cancer positive subjects. Somatic alterations were detectable in the plasma in 7 of 11 cancer-positive subjects. No somatic alterations were detected in the plasma from healthy subjects.

Subject Information		Test Status		Test Results Detail		Tumor Biopsy					Blood Sample	
Subject ID	Primary Diagnosis	Tumor	Blood	Detected Variant Type	Variant Detail (SNV Only)	1	2	3	4	5	Pre Surgery	Post Surgery
PT01	Cystic Renal Carcinoma	Positive	Positive	SNV	PDGFRA (c.2140+28C>T)	●	●	●	○	◌	N/A*	○
				SNV	ENSCAFG00000047557 (p.LEU117GLN)	○	○	●	○	◌	N/A*	○
				CNV		●	●	●	●	◌	●	○
PT02	Cholangiocellular Carcinoma	Positive	Positive	SNV	PDGFRB (p.Leu583Pro)	●	○	○	○	◌	○	◌
				SNV	EGFR (p.Leu805Arg)	○	○	●	○	◌	○	◌
				SNV	CTNNB1 (p.Lys335Ile)	○	○	○	○	◌	●	◌
				CNV		○	○	●	●	◌	●	◌
PT03	Matastatic Pancreatic Carcinoma; Hepatocellular Carcinoma; Metastatic Splenic Hemangiosarcoma	Positive	Positive	SNV	NRAS (p.Gly13Arg)	○	○	●	○	●	●	○
				SNV	TP53 (p.Gly318Arg)	○	●	○	○	○	●	○
				SNV	KRAS (p.Gly106Asp)	○	○	●	○	○	○	○
				SNV	TP53 (p.Arg332Ile)	○	○	●	●	○	●	○
				SNV	PTPN11 (p.Gly507Val)	○	○	○	○	○	○	●
				CNV		●	●	●	○	●	●	●
PT04	Anal Sac Adenocarcinoma	Positive	Positive	CNV		●	●	●	●	◌	●	◌
PT05	Bilateral Anal Sac Adenocarcinoma	Positive	Negative	CNV		●	●	○	●	◌	○	◌
PT06	Multifocal Mast Cell Tumor	Positive	Negative	CNV		●	●	○	◌	◌	○	◌
PT07	Anal Sac Adenocarcinoma	Positive	Negative	SNV	PIK3CA (p.Tyr1021Cys)	○	○	●	◌	◌	○	◌
				CNV		●	○	○	◌	◌	○	◌
PT08	Soft Tissue Sarcoma	Positive	Positive	CNV		●	○	◌	◌	◌	●	◌
PT09	Multifocal Soft Tissue Sarcoma	Positive	Negative	CNV		●	●	●	●	●	○	○
PT10	Osteosarcoma	N/A	Positive	SNV	CALR (c.1054-37G>A)	◌	◌	◌	◌	◌	●	◌
				SNV	TP53 (p.Glu71SerfsTer7)	◌	◌	◌	◌	◌	●	◌
				CNV		◌	◌	◌	◌	◌	●	◌
				Fragment Length		◌	◌	◌	◌	◌	●	◌
PT11	Hemangiosarcoma (renal, splenic)	N/A	Positive	SNV	PIK3CA (p.Asn347Lys)	◌	◌	◌	◌	◌	●	◌
				Fragment Length		◌	◌	◌	◌	◌	●	◌
PT12**	Presumed cancer-free	N/A	Negative	None		◌	◌	◌	◌	◌	○	◌
PT13**	Presumed cancer-free	N/A	Negative	None		◌	◌	◌	◌	◌	○	◌
PT14**	Presumed cancer-free	N/A	Negative	None		◌	◌	◌	◌	◌	○	◌
PT15**	Presumed cancer-free	N/A	Negative	None		◌	◌	◌	◌	◌	○	◌
PT16**	Presumed cancer-free	N/A	Negative	None		◌	◌	◌	◌	◌	○	◌

**The baseline blood sample for PT01 failed minimum coverage requirements.*

***Subjects PT12-PT16 were presumed to be cancer-free due to no history of cancer and no clinical signs consistent with cancer at the time of blood collection.*

CNV = Copy Number Variant; SNV = Single Nucleotide Variant; NA = Not Applicable

LEGEND

● Variant detected

◌ Sample at given timepoint not available

○ No variant detected
