# Supplementary figures and images for: Blood-Based Liquid Biopsy for Comprehensive Cancer Genomic Profiling Using Next-Generation Sequencing: An Emerging Paradigm for Non-invasive Cancer Detection and Management in Dogs
Source: Front Vet Sci. 2021 Jul 8;8:704835. doi: 10.3389/fvets.2021.704835 (PMC8297996; doi:10.3389/fvets.2021.704835)

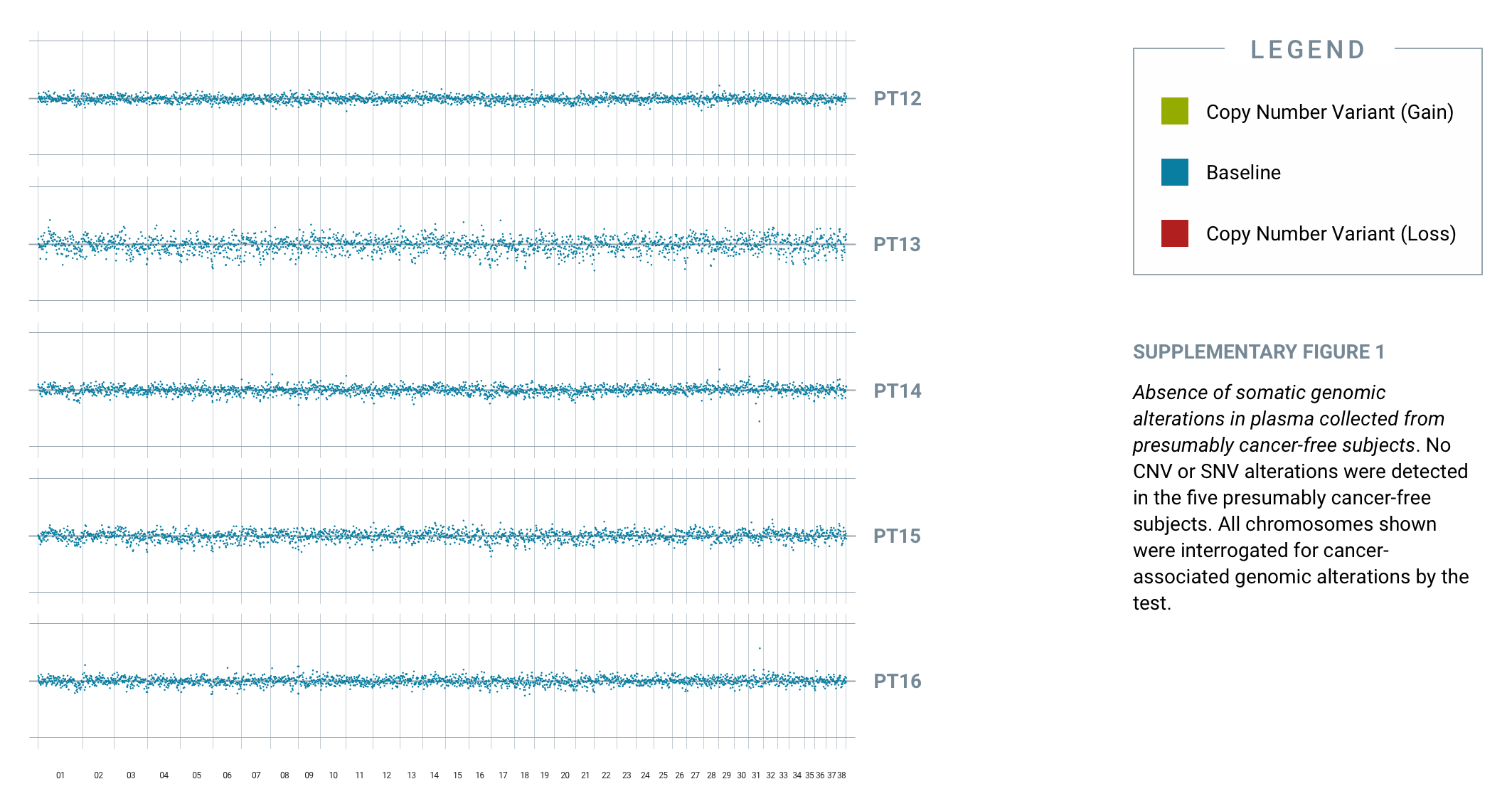

Supplement: Supplementary file 2 [file Image_1.TIFF]
